# An Evaluation of the Impact of Monetary Easing Policies in Times of a Pandemic

**DOI:** 10.3389/fpubh.2020.627001

**Published:** 2021-01-14

**Authors:** Ying Li, Yunpeng Sun, Mengya Chen

**Affiliations:** ^1^School of Economics, Tianjin University of Commerce, Tianjin, China; ^2^Portsmouth Business School, University of Portsmouth, Portsmouth, United Kingdom

**Keywords:** COVID-19 outbreak, monetary policy, event-study analysis, CPI, real GDP

## Abstract

This article tests five major economies of the world, United Kingdom, Japan, Brazil, Chin and lastly, India, for the changes in the monetary policy decisions that have been implemented following the Covid-19 outbreak. The assessment was undertaken in the form of an event study analysis, further substantiated with a regression analysis conducted for exploring the significance of CPI and real GDP in predicting the policy interest rates in the economy. The results of the event study analysis presented that the abnormal changes in the interest rates were statistically significant in the case of the United Kingdom, Brazil, and China, while the abnormal changes were found to be statistically insignificant in the case of India and Japan.

## Background of the Study

Monetary policy can be explained as the decisions and actions undertaken by the central banks to manage money supply and availability of credit in the economy. A majority of the economies, in the present-day scenario, make use of the monetary policy initiatives to foster economic growth and propel economic momentum. Among the top monetary policy tools available for the policymakers is the management of the interest rates in the economy. Policy interest rates are used to control the supply of money in the economy, in the sense that as the interest rates increase in the economy, the supply of money is limited which limits the demand of money as the acquisition of funds becomes more expensive (1). As there are inflationary pressures in the economy, the monetary policy theory dictates that the interest rates are decreased by the policymakers to enhance the production capacity and increase in the aggregate supply (2). However, inflationary and deflationary pressures in the economy are a part of the cyclical fluctuations. However, in a series of unprecedented events, the normal business cycles are disrupted by the occurrence of unprecedented events and crises which shock the normal economic flow, which bring the economic variables to a sudden standstill. The erratic consequences of the economic shocks include changes in the consumer price index or inflation and the production, aggregate supply and consumption demand in the economy (3).

Monetary policy tools, both conventional and unconventional, endeavor to bring about price stability in the economy while enabling economic growth and development. The conventional methods for controlling the monetary base in the economy entail controlling for lending and bank interest rates in the economy (4). As the economy is faced with choppy waters, the policymakers try and ensure normalcy by giving a slight push to the industries by lowering the interest rates and easing credit availability in the economy (5). This ensures that the productive capacity is brought at par, and the output gap within the economy narrows down. On the consumption end, releasing credit availability allows an increase in the consumption demand as credit availability increases the aggregate supply, lowering the general price level in the economy. This hands-on approach toward the management of credit availability allows the policymakers to maintain financial and economic stability in the face of the cyclical economic fluctuations. However, as discussed, there are several unforeseen errants or shocks, which might affect the economic operations.

Considering to the shortage of research studies examining effects of monetary policy on main economies' development in Covid-19 as well as the scarcity of the existing literature on the relationship between monetary policy with the pandemic, this study is motivated to contribute to the expansion of the existing literature on this topic by providing an evidence from the effects of monetary policy in different countries under the Coronavirus pandemic. It aims to clarify how different economies' monetary policy are how to affect the real economy and price level within the Coronavirus pandemic. The Covid-19 pandemic is one such unprecedented event which has shaken the world economy (6). Starting off with the news associated by with viral outbreak in China began surfacing, and the world bank issued guidelines for undertaking preventive measures against the spread of the virus (7). These safeguards include the national shutdowns, import restrictions and travel restrictions which have limited the growth and productive capacity of the countries. The current article delves on the impact of the coronavirus outbreak on the monetary policy on the major world economies of the United Kingdom, Japan, Brazil, China, and India.

## Review of Literature

The novel coronavirus originated in China has been declared as an emergency pandemic situation having a significant impact on the economies all over the world. The virus has impacted the output yields and investments in all industries as well as consumption among households. Generally, the policymakers, in order to contain the impact of the pandemic, use monetary policies to affect the funds available in the financial system which in turn affects the interest rate and eventually the asset prices. With the change in interest rates and asset pricing, the consumption and investment pattern changes, allowing the economy to sustain and grow. The current study discusses the impact of Covid-19 pandemic on the consumption, manufacturing, and investment in the economies of Germany, UK, and France and the influence of monetary policies of the central banks in curtailing the impact of the pandemic.

### Sudden Stops and Their Impact on the Economy

Sudden stops have been studied in the empirical and theoretical literature since the late 1990s. In the times of globalization and high capital mobility, sudden stops have become a major issue in economics and international finance. The focus on sudden stops has been increased after the Global Financial Crisis of 2007–2008 with a growing literature reviewing the role of macroprudential regulation in obviating financial crisis. Sudden stops are economic fluctuations characterized by a sudden contraction or loss of capital inflows and associated irregularities due to reduced access to international financial markets. The economic term' sudden stops' was coined with the abrupt drying up of capital inflows during the Mexican crisis of 1994. The phrase was then referred in many following crises like an Argentine crisis (1995), Russian crisis (1998), the Brazilian crisis (1999), and others (8). Sudden stops are often associated with currency crashes as it results in the fall of the foreign exchange rate.

The awareness about capital inflow volatility and reversals has increased in the policy community based on which International Monetary Fund (IMF) has developed new and more sympathetic capital control as well as international capital markets intervention policies. An example of a sudden stop is the “taper tantrum” of 2013 when Federal Reserve was expected to taper its purchase of securities which would have resulted in a market crash with a rise in US interest rates leading to capital outflow from emerging economies. This suggests that sudden stops are growing disruptive. These sudden stops have real and financial implications. The financial effects occur first with depreciation in the exchange rate, a decline in reserves, and a fall in equity prices. It also leads to a deceleration in GDP growth, a slowdown in investment, and strengthening of the current account. In the first four quarters of the sudden stop, the GDP falls by 4% year on year (9). GDP declines even faster in the second subperiod demonstrating a global level shock. These financial effects can only be partially offset using macroeconomic positions.

In the 1990s, the countries responded to sudden stops by reducing the exchange rate, floating currency, and following the new exchange rate, or implement a tighter monetary policy as policy responses. These countries, in the worst-case scenario, also resort to IMF for aid in the form of fiscal tightening, trade reforms, and privatization of public enterprises (10). However, in the second phase, even less tightening of monetary and fiscal policy work. In order to support economic activity and capital markets, some companies resort to reducing policy interests. With currency depreciation, little monetary stringency would work as countries had lowered mismatches in foreign currency, reducing damage in balance sheet due to depreciation. The impact of sudden stops differs for a country with a fixed foreign exchange rate policy than for a country with a flexible foreign exchange rate policy. This is because a country with fixed exchange rate regime is not able to offset the reduction in demand through expansionary monetary policy or achieve exchange rate adjustment with the help of nominal depreciation (11). Countries with stronger fiscal balance are able to deal with the sudden stops with minimum fiscal consolidation. In the 2000s, resorting to IMF for aid was reduced as countries had accumulated a reserve of international currency and moved to flexible exchange rates.

**Figure 1:**
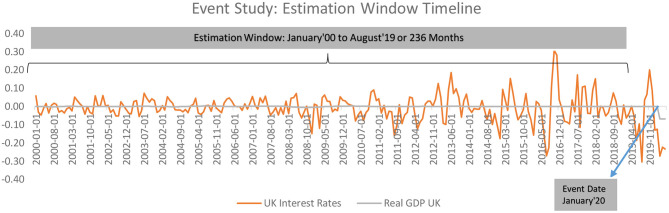
Event timeline: Covid-19 pandemic.

Emerging markets have been experiencing the impact of sudden stops despite flexible exchange rates, stronger fiscal budgets, stronger financial markets, and less foreign currency differentiation. The occurrence of these sudden stops have not reduced, and any benefit from stronger fiscal and monetary positions of countries does not offset the sudden stops coming from different countries. The progress on the fiscal and monetary policies have also not reduced the implications of the stops in a significant manner (12). The output reduction in the first quarter is slightly higher than the second subperiod. With increased globalization and transaction in international financial markets, countries experience capital flow reversal more often due to sudden stops and these reversals have unruly output impacts (9). It is disturbing that neither the national officials with their monetary or fiscal policies nor the international financial institutions with their increased new financial facilities have helped in reducing the impacting of sudden stops in the emerging markets. The frequency and severity of sudden stops remain the same in all subperiods. The decline in GDP second subperiod as the capital inflows in the preceding subperiod is higher and large capital reversals are experienced when the sudden shock shift to the second subperiod. During the sudden stops, changes are observed in the global economic conditions and policies and characteristics of affected countries. Stronger policies, however, had an impact at the national level, as shown in Figure 1. During sudden stops, it is essential for the emerging countries having high budget deficits and high inflation rate to tighten fiscal and monetary policies. According to Efremidze et al. (13), sudden stops have a negative impact on financial deficits and require governments to take painful steps to reduce the effect on financial markets. Paradoxically, exchange rate policy, tightening of monetary, and fiscal policies as a response to the stops have been found to be effective at a national level, but at the global level, the reversal of international capital flows enhances the effect of external stops. Low trade openness and low financial globalization reduces the possibility of a spillover of a sudden stop and also minimize the impact of sudden stops on the countries.

### Monetary Policy and Fiscal Stability in Pandemic Times

Covid-19 has given an economic shock to countries across the globe with its alarming speed and gigantic magnitude. This led to steep recessions in many industries across countries. Despite the global policy support, the pandemic is estimated to impact the global economy in the form of a 5.2% dip in the GDP in 2020, the highest in eight decades. The per capita incomes of emerging and developing economies have been contracted by far this year. The impact on the global economy would worsen if the pandemic took longer to get controlled and financial stress persists. The pandemic highlights the need for economic actions apart from urgent health-related policy actions to reduce the vulnerable consequences and improve the capacity of countries' system of dealing with the pandemic in future. Severe outbreaks in these countries result in international spillovers and negative impact on the global value chains and financial markets. GDP growth of regions such as Latin America, Central Asia, and Europe has been contracted due to international spillover from South Asia's GDP downgrade and the domestic outbreak of the pandemic resulting in lockdown measures. Many countries mitigated the impact of the pandemic by adopting strict fiscal and monetary policies. Per capita income is also expected to downgrade in 2020 in all emerging and developing economies. Covid-19 will leave lasting scars to the economies creating a devastating impact on the already fragile developing economies.

Monetary and fiscal policy measures can mitigate the short-term impact of the pandemic on the economy and productivity while comprehensive reforms would be required to minimize the long-term impact of the pandemic on the growth of the nation by improving governance, public health policies, and general business environment. The Covid-19 outbreak has led to a collapse in demand for oil, an upwelling oil inventories and the lowest decline in oil prices. In the initial stage of the pandemic, with all the lockdown restriction, oil prices cannot buffer the impact of Covid-19, but it can certainly help the economy to recover once the restrictions are lifted. The fiscal positions of energy-exporting emerging economies have been strained for a while now, and the pandemic has resulted in a collapse in their oil revenues. Thus, fiscal policy needs to be implemented for a sustainable economic position in a country. Recently, a sharp rise in the number of virus patients has been witnessed even in the developed economies let alone emerging and developing economies. The second wave of infection has resulted in the loss of income, trade, and investments. In these scenarios, the government fiscal policies and central bank's monetary policies are likely to renew the collapsed consumption of households. The low income restrained the borrowing capacity of households, and therefore, they could not maintain the consumption. The loose monetary policy would provide liquidity and purchasing power to the households to maintain their basic level of consumption despite a low level of savings. The ability of monetary policy and welfare systems to reduce income losses differ from country to country and is generally lower in low-income countries. The domestic investment comes to a halt during uncertainty like pandemics, and the outputs worsen. Restrictions imposed due to COVID-19 reduce the ability of fiscal and monetary policies to limit the consequences of the pandemic. Businesses are affected due to lack of demand, shortage of raw material, and costs of providing safety to employees from the virus. In some sectors, even fiscal stimulus is ineffective as the processes are completely shut (6). In such cases, the global recession is expected with high fiscal debts for low-income countries.

### Role of Central Banks and Effect of Interest Rates: Delphic and Odyssean Monetary Policy Theory

The output and aggregate demand in an economy are not only influenced by the monetary policy, but interest rates also play a major role. Since interest rates have an impact on the economic policies and decisions, information revealed by the central banks about the future rates is an important medium based on which the monetary policy influence the macroeconomy.

Such impact reveals that central banks can control the aggregate demand by expressing that interest rates will remain low for a long time when the policy rate cannot be lowered due to effective lower bound (ELB). Other than the planned money supply injection and interest rates influence, sudden stops, and natural occurrences also have an influence on the yield curve. A piece of bad macroeconomic news impacts the yield curve, and as a reaction to it, the central bank needs to adjust its monetary policy. There are generally two kinds of surprises that have a macroeconomic impact on the central bank policy. As discussed by Campbell et al. (14), one of these sudden stops or natural occurrences has been termed as “Delphic” shocks as it explains the reaction of the central bank to the bad macroeconomic news by giving an oracle. The second type of shock is termed as “Odyssean” shock in which the central bank “ties its hands to the mast” by promising to twig to the announced plan for the interest rate. Central banks provide Delphic and Odyssean information even though guidance policies have been implemented. It is generally observed that Odyssean surprises are effective in improving aggregate demand and output in the economy. A central bank announcement has a Delphic nature if it raises interest rates and creates positive inflation expectations. When the inflation expectations reduce as a result of central bank announcements, Odyssean shocks occur. These announcements from a central bank not only makes the investors pessimistic or optimistic about the future of their investment, but the existence of these two shocks also helps in analyzing the response of financial and economic variables to monetary policy expectations (15). D'Amico and King (16) explained the variations and expectations of variables interest rates, output and inflation using VAR. They imposed different sign restrictions on short-term interest rate pattern as well as tracked expected inflation and GDP. This sign imposition allowed the authors to identify the domination of Delphic guidance on the Odyssean pattern

### Research Gap

A wide literature discussing the impact of sudden stops such as financial crisis or pandemics on the economy and suggesting the importance of monetary easing policies in boosting money supply in the economy during the times of crisis is available. However, the specific impacts of monetary easing policies on curtailing the pandemic effects are limited specifically for the economies such as Germany, France, and the United Kingdom. Thus, the present study aims to critically assess the short-term effects of monetary easing policies on the money supply in the economy especially during the times of pandemic with consideration on the recent scenario of Covid-19. Majority of the studies have discussed the impact of monetary and fiscal policies on the economy using the VAR model. Our study would adopt a mixed methodology to answer the research questions in the context of the recent Covid-19 crisis, discussed in the following section.

## Methodology

This section describes the research methodology used to answer the research questions. It explains the data and variables used and the analysis method adopted to conduct the research. It also details out the measures and methods used for analyzing data, the findings and results of which have been discussed in the following section.

### Data Description

The study analyses the macroeconomic variables and financial factors of the countries UK, China, Brazil, India, and lastly Japan to evaluate the role of monetary policies in curtailing the pandemic consequences and in aiding the recovery of the economy. The monetary policies involve fixing a refinancing operations rate that is unique for every country. The study analysis considered variables with the capability of measuring economic activity such as inflation rate measured using Consumer Price Index (CPI) and real GDP. The data for real GDP was available in the form of an index with 2015 as the base year, harmonized for each of the countries being examined.

The quantitative easing in unconventional monetary policy is expected to raise inflationary pressures in the economy, which, in turn, impact the prices. The higher inflation rate is expected to increase current expenditure because of higher expected inflation, and thus, the price level also increases. As studied by scholars, the monetary policy has the capability to reduce the negative consequences of the pandemic by improving consumption and investment patterns. The current study, therefore, analyses the impact of the federal fund rate on the interest rate and real GDP of an economy during the times of pandemic Covid-19.

### Variables and Measures

The study uses the monetary policy premise, as discussed by Taylor (17) in his study. It explains that the interest rates prevailing in an economy are a result of the real income estimates intruded by the monetary policy refinancing operations. Based on the analysis conducted by Taylor (17) in his paper “*Discretion vs. policy rules in practice*,” the current study aims to find the relationship between federal funds rate and macroeconomic variables such as inflation rate and real GDP using a predefined equation model. The study has been conducted in the context of UK, China, Brazil, India, and lastly Japan's monetary policy determining the inflation rate to curb the negative impact of the pandemic and boost aggregate demand and consumption through inflation rate moderation. The data period under study ranges from the fourth quarter of 2019 (beginning of the pandemic Covid-19) to the third quarter of the year 2020 (current period). The variables under study are as follows:

1. Real GDP (deviation from the target)
2. Federal Funds Rate in percentage
3. The inflation rate over the past four quarters.

The Taylor's Rule applied in the Taylor (17) study can be reiterated using a model equation as presented below:

$$\begin{array}{l} r=p+0.5y+0.5\;\left(p-2\right)+2 \end{array}$$

Where

P denotes inflation rate, computed using the average Consumer Price Index (CPI) for the past 4 months;

Y represents the real GDP which is the per cent deviation from the target GDP set by the policymakers or monetary authorities.

Although the size of the coefficients can vary because of lack of consensus, the current study uses a representative policy rule of the countries under consideration. The rule states that the federal fund rate increases in case the inflation rate rise above the 2% target or the real GDP increases above the trend GDP. The federal fund rate would be twice if the inflation rate and real GDP matches the target.

The analysis would present the descriptive statistics for the above-discussed variables as well as to conduct a trend analysis of how these macroeconomic variables have changed over the past four quarters due to the occurrence of the pandemic.

### Methods

#### Regression Model

The empirical model used Taylor's model to examine the relationship between the federal funds rate and the macroeconomic variables such as real GDP and inflation rates in the context of the United Kingdom, Japan, Brazil, China, and India. The figures of the past 20 years have been considered as they would reflect the pandemic situation and impact of monetary policies of the government in controlling the pandemic situation. The study uses a quantitative model, and the data has been collected through reliable secondary sources. The secondary sources used for data collection are FRED Economic Research database which has reliable data for the past 20 years (18). It is a digital library with a compilation of data related to economic and financial factors, and public access is available for a million data points. The reliability of the data source comes from its publication by the Federal Reserve Bank of St. Lois Review.

The model equation used in this context has been presented as follows.

$$\begin{array}{l} Federal\;Funds\;Rate=\alpha +\beta 1\;\left(real\;GDP\right)+\beta 2\left(inflation\;rate\right) \end{array}$$

The equation represents the federal funds rate as a function of the macroeconomic variables (real GDP and the inflation rate). The analysis process would start off with the assessment of the factors affecting the monetary policy interest rates in the real-world scenario. Starting off with Taylor's monetary policy rule formula for computing the federal funds rate, we take a standpoint for examining the nature and direction of the factors that a make up the policy interest rates. This has been undertaken with the help of estimating a series of regression models for the five countries under consideration. The data has been analyzed using Microsoft Excel, and the results of the same have been presented in the following section.

#### Event Study Methodology

To examine the impact of WHO announcing the Covid-19 pandemic on the monetary interest rates and the real GDP using the event study methodology. This particular methodology has been used in a large number of studies to decipher the impact of the announcement of an event on the value of a firm or for a policy interpretation (15, 19). Making use of the event study methodology is that it encapsulates the short-term impact of a given economic/ financial or even business events. In the current scenario, the event study analysis has allowed us to assess the monetary policy impact caused by the coronavirus outbreak as instigated by the changes in the real GDP due to the event.

**Figure 2:**
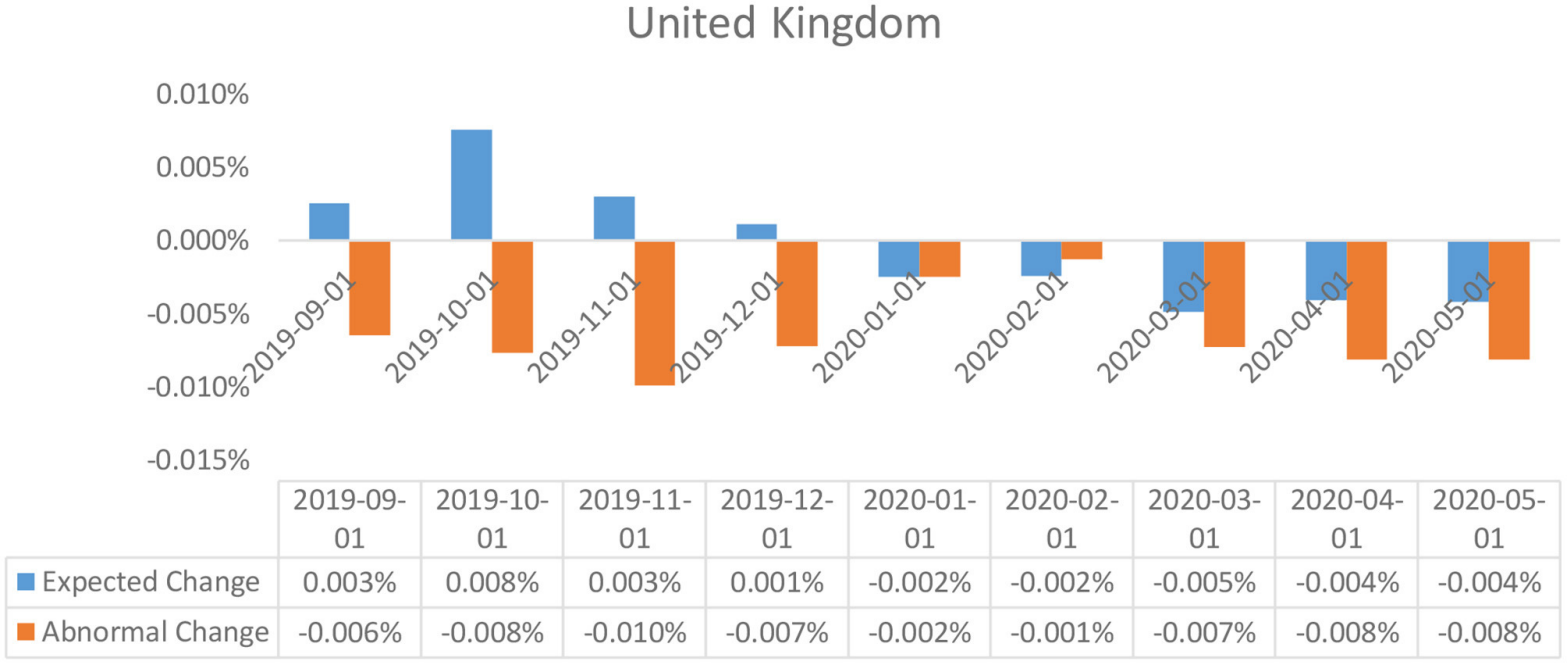
Abnormal changes in policy interest rates over the event window: United Kingdom.

**Figure 3:**
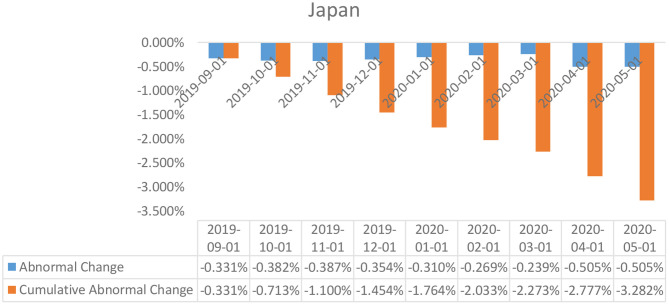
Abnormal changes in policy interest rates over the event window: Japan.

**Figure 4:**
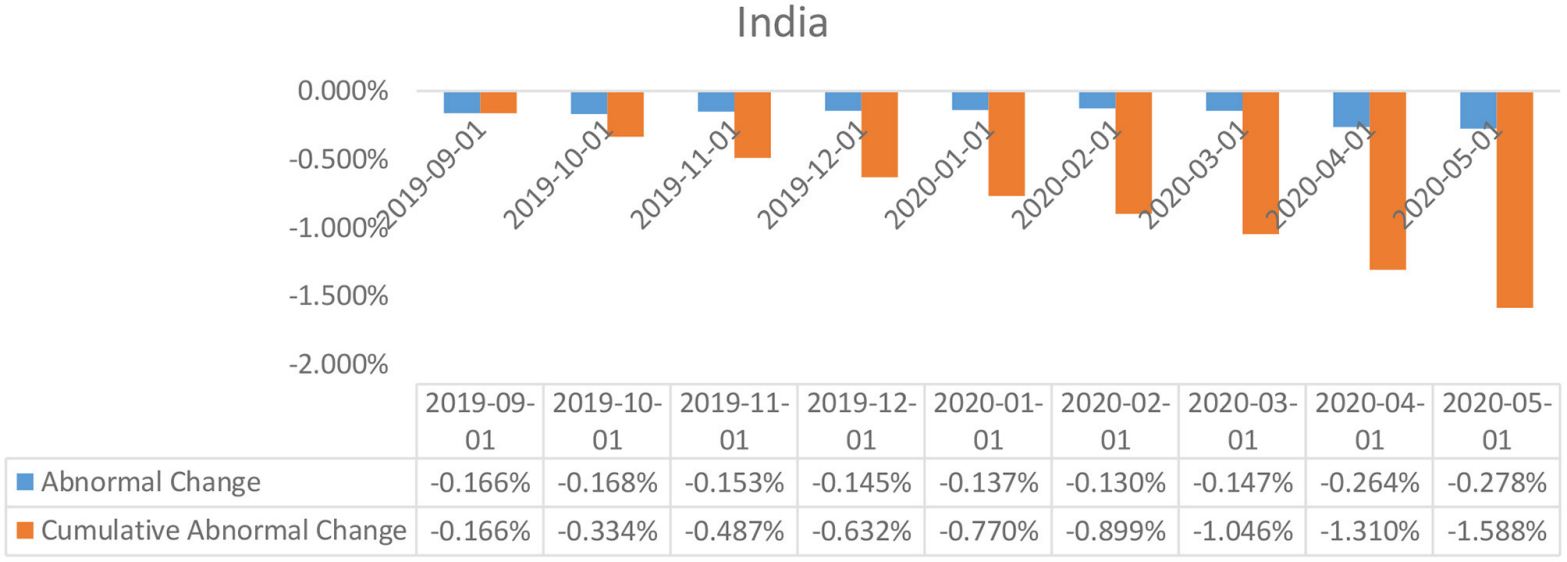
Abnormal changes in policy interest rates over the event window: India.

**Figure 5:**
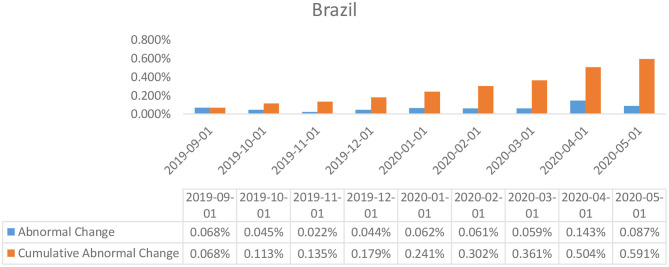
Abnormal changes in policy interest rates over the event window: Brazil.

**Figure 6:**
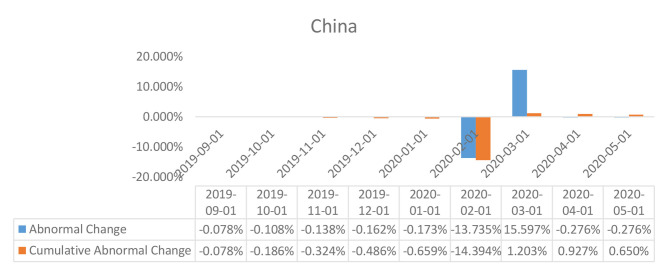
Abnormal changes in policy interest rates over the event window: China.

The Covid-19 pandemic struck the global landscape sometime around later November to early December 2019. However, the WHO issued a series of guidelines for the world leaders regarding the safeguards against the spread of the coronavirus pandemic on 10 January 2020, which has been taken as a guideline for the event declaration (20). The impact of the global pandemic can be seen through a slump in the economic activity worldwide through the nationwide shutdowns enforced by the governments and the policymakers (21). This, in turn, has presented a decrease in the aggregate demand and aggregate output in the economy and thereby a slump in the real GDP in various parts of the world. The impact of the pandemic outbreak, as seen on the macroeconomic indicators like the output levels in the economy has also been mitigated by the policymakers through changes made in the monetary policy response packages. This has been examined through event study analysis for the impact of the Covid-19 event on the economies of the United Kingdom, Japan, China, India and lastly, Brazil (as shown in Figures 2–6).

**Figure 7:**
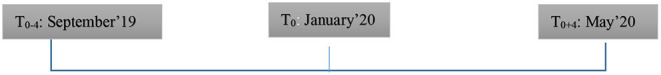
Event window.

The estimation and time window can thus be illustrated as;

Here, as can be seen, the estimation window has been constructed for 236 months ranging from 01 January 2000 till 01 August 2019, comprising of 236 months in Figure 7. The estimates have been developed as a monthly change in the value of the policy interest rates in the current period over the preceding time period.

$$\begin{array}{l} V=\frac{V1-V0}{V0} \end{array}$$

Where, V1 is the value of the estimate in the current time period, and V0 is the value in the preceding time period while V represents the value estimated as a monthly change in the values observed for the policy interest rates and real GDP.

The event study methodology empirically entails the assessment of the abnormal changes (increase or decrease) of performance variables upon the announcement of a particular event. The abnormal changes in the policy interest rates have been estimated every month of the event window ranging from September 2019 till May 2020 has been estimated as;

$$\begin{array}{l} AC_{i,t}=OC_{i,t}-TC_{i,t} \end{array}$$

Where in,

*AC*_*i,t*_ represents the abnormal changes in the policy interest rate “i“ in the month “t;”

*OC*_*i,t*_ represents the observed changes in the policy interest rate “i” in the month “t,” over the length of the event window;

And *TC*_*i,t*_ represents the theoretical changes in the policy interest rate “i“ in the month “t,” over the length of the estimation window.

The theoretical variations in the policy interest rates of each of the countries have been estimated as a consequence of the regression equation comprising of the policy interest rate as the dependent variable and the real GDP as the independent variable.

$$\begin{array}{l} {Changes\;in\;Interest\;Rates}_{i,t}=\alpha +\beta \left({Changes\;in\;Real\;GDP}_{i,t}\right) \end{array}$$

Deriving a slope and the intercept values for the real GDP and the interest rates allows us to draw a theoretical value for the changes in the interest rates as instigated by the changes in the real GDP in the economy. Once the observed changes and theoretical changes in the policy interest rates have been categorized, we move on to the assessment of the observed abnormalities in the policy interests in the countries under consideration. This has been undertaken with the help of the application of a cross-sectional Student's *t*-test. The estimation formula used for the same has been presented below;

$$\begin{array}{l} {Student^{\prime }s\;T\;Test}_{t}=\frac{{Cumulative\;Abnormal\;Change}_{t}}{Standard\;error\;of\;the\;TC\;} \end{array}$$

Where in the cumulative abnormal changes refer to the abnormal changes in the policy interest rates for every month passed in the event window. The formula can also be presented as;

$$Cumulative\;Abnormal\;Change_{t}=\frac{\sum_{i=1}^{N}AC_{i,t}}{N}$$

Where N is the length of the event window. Next, the standard error of the theoretical changes has been estimated using the STEYX function of the excel and can be theorized by the following formula;

$$\begin{array}{l} Standard\;Error=\frac{\sum {\left(Yi-Yi^{\prime }\right)}^{2}}{n-2} \end{array}$$

Where in, Yi represents the values for Observed Changes and Yi' represents the predicted values or the theoretical changes in the policy interest rates. The estimated values for the *t*-test have then been used to examine the statistical significance of the abnormal changes observed in the policy interest rates in the case of each of the five countries being examined.

## Empirical Results

### Regression Estimates

Starting with the assessment of the impact of the irregularity represented by the spread of the Covid-19 pandemic on the economies all across the world, and in order to deliver robust results for the assimilated data, we conduct a regression analysis in order to examine the factors affect the policy interest rates in an economy. Drawing inspiration from Taylor's Rule, we conduct a regression analysis with the policy interest rates as a dependent variable and real GDP and inflation as the independent variables. For each of the countries, the following regression equation has been computed;

$$\begin{array}{l} {Policy\;Interest\;Rate}_{i}=\alpha +\beta_{1}\left(CPI_{i}\right)+\beta_{2}\left({Real\;GDP}_{i}\right)+\varepsilon \end{array}$$

Where,

The exchange rate is the policy interest rate

CPI is the consumer price index

Real GDP, as acquired from the open access sources reflects on the index of real GDP I country i

ε is the residual noise

α is the intercept.

The results of the regression analysis conducted have been summarized in Table 1.

**Table 1:** Regression model.

	**UK**	**Japan**	**India**	**China**	**Brazil**
Intercept	7.9384^**^	−7.2397^**^	20.3858^**^	−4.3242^**^	159.5891^**^
	[4.8021]	[−7.4804]	[3.5698]	[−2.2484]	[9.7853]
CPI	−0.1265^**^	0.0123^**^	0.0085^**^	−0.0030^*^	−0.1164^**^
	[−47.4029]	[2.2820]	[3.4107]	[−1.7095]	[−14.2460]
Real GDP	0.0675^**^	0.0633^**^	−0.1421^**^	0.0768^**^	−1.3163^**^
	[4.2273]	[7.8101]	[−2.4873]	[3.9809]	[−8.0939]
R square	0.9081	0.2144	0.0675	0.0687	0.5146

** *Implies that the probability value of the t-statistic of the regression coefficient is less than 0.05, making the coefficient statistically significant at 95% level of significance*.

* *Implies that the probability value of the t-statistic of the regression coefficient is less than 0.10, making the coefficient statistically significant at 90% level of significance*.

The results from Table 1 indicate that both the regressor have a statistically significant impact over determining the policy interest rates in each of the countries under consideration, United Kingdom, Japan, Brazil, China, and India. However, the nature and direction of the relationship between the independent and dependent variable varied in each of the cases. In the case of the UK, while the real GDP index had a positive relationship with the policy interest rate, CPI had a negative relationship with the interest rates in the economy. This, however, is in line with the theoretical implications of the monetary policy theory, that inflation (represented by the consumer price index in the economy) has an inverse relationship with the interest rates and real GDP has a direct relationship with the interest rates (22). Similar results were reported in the case of the Chinese economy wherein the CPI had an inverse relationship with the interest rates and real GDP had a positive relationship with the dependent variable. In the case of the Japanese economy, both CPI and real GDP had a positive impact on interest rates. On the other hand, in the case of the Indian economy, real GDP had a negative impact over the interest rates while CPI had a positive impact on the interest rates, which contrasts the inference of the model presented in the case of the UK. Lastly, in the case of the Brazilian economy, it was observed that both CPI and real GDP had an inverse relationship with the policy interest rates observed.

Overall, the findings from the regression models constructed for the five economies under consideration present sufficient support for the explanatory variables, i.e., CPI and real GDP having a significant impact over the policy interest rates, as being helpful while determining the future trends of the interest rates for the economies. This comes in especially helpful in examining the impact of the Covid-19 pandemic on the real GDP in the economy and the resultant impact on the policy interest rates (23–25).

### Event Study Results

The results of the event study analysis carried out for United Kingdom, Japan, Brazil, India, and China over the length of the given estimation window have been summarized in Table 2 (for UK, Brazil, and Japan) and Table 3 (for India and China). The assessment of the abnormal changes in the interest rates, egged against the cumulative interest rates reflects on a significant downward movement for both the estimates for the United Kingdom, Japan, and India. This can be explained by a constant deterioration in the observed values of interest rates by the British and Indian policymakers as a response to the Covid-19 outbreak. Also affecting the estimates are the deteriorating real GDP estimates for each of the economies in the first 3 months of 2020, which was a direct consequence of the widespread government-imposed nationwide shutdowns. Peculiar observations can be seen in the case of the policy interest rates abnormalities in the case of China, wherein, despite being the worst hit epicentre of the coronavirus outbreak, the policymakers did not resort to monetary relaxations, and no particular changes were observed in the policy interest rates. However, the Chinese economy did suffer considerably, owing to the limited production activities underway due to the shutdowns.

**Table 2:** Estimation window: the United Kingdom, Brazil, and Japan.

	**United Kingdom**				**Japan**				**Brazil**			
**Date**	**Expected change (%)**	**Abnormal change (%)**	**Cumulative abnormal change (%)**	**AR *T*-test**	**Expected change (%)**	**Abnormal change (%)**	**Cumulative abnormal change (%)**	**AR *T*-test**	**Expected change (%)**	**Abnormal change (%)**	**Cumulative abnormal change (%)**	**AR *T*-test**
2019-09-01	0.003	−0.006	−0.065	−0.48092	0.003	−0.331	−0.331	−1.65109	0.026	0.068	0.068	0.326262
2019-10-01	0.008	−0.008	−0.014	−0.57052	0.003	−0.382	−0.713	−1.90376	0.045	0.045	0.113	0.216793
2019-11-01	0.003	−0.010	−0.240	−0.73541	0.003	−0.387	−1.100	−1.92852	0.067	0.022	0.135	0.106298
2019-12-01	0.001	−0.007	−0.312	−0.53604	0.003	−0.354	−1.454	−1.76777	0.046	0.044	0.179	0.212715
2020-01-01	−0.002	−0.002	−0.336	−0.18419	0.003	−0.310	−1.764	−1.54647	0.030	0.062	0.241	0.301054
2020-02-01	−0.002	−0.001	−0.349	−0.09614	0.003	−0.269	−2.033	−1.34078	0.033	0.061	0.302	0.2967
2020-03-01	−0.005	−0.007	−0.122	−50.4693	0.003	−0.239	−2.273	−1.19405	0.038	0.059	0.361	0.283356
2020-04-01	−0.004	−0.008	−0.937	−50.7862	0.003	−0.505	−2.777	−2.51666	0.048	0.143	0.504	0.690663
2020-05-01	−0.004	−0.008	−0.750	−50.7747	0.003	−0.505	−3.282	−2.51666	0.104	0.087	0.591	0.420762

**Table 3:** Estimation window: China and India.

	**China**				**India**			
**Date**	**Expected change (%)**	**Abnormal change (%)**	**Cumulative abnormal change (%)**	**AR *T*-test**	**Expected change (%)**	**Abnormal change (%)**	**Cumulative abnormal change (%)**	**AR *T*-test**
2019-09-01	0.004	−0.078	−0.078	−0.72768	−0.011	−0.166	−0.166	−1.13455
2019-10-01	0.004	−0.108	−0.186	−1.00997	−0.004	−0.168	−0.334	−1.14898
2019-11-01	0.004	−0.138	−0.324	−1.28823	−0.011	−0.153	−0.487	−1.05058
2019-12-01	0.004	−0.162	−0.486	−1.51871	−0.011	−0.145	−0.632	−0.99481
2020-01-01	0.004	−0.173	−0.659	−1.61524	−0.011	−0.137	−0.770	−0.93844
2020-02-01	0.004	−13.735	−14.394	−128.414	−0.011	−0.130	−0.899	−0.8872
2020-03-01	0.004	15.597	1.203	145.8241	0.012	−0.147	−1.046	−1.00322
2020-04-01	0.004	−0.276	0.927	−2.58472	−0.011	−0.264	−1.310	−1.80783
2020-05-01	0.004	−0.276	0.650	−2.58472	0.003	−0.278	−1.588	−1.90524

In the case of the United Kingdom, based on the *t*-test for the abnormal changes, the abnormal changes in the interest rates were statistically significant after the event announcement in the post event window. And thus, it can be considered that the policy interest rates in the United Kingdom were significantly impacted by the changes in the values for Real GDP as triggered by the Covid-19 pandemic outbreak.

In the case of Japan, the abnormal changes in the policy interest rates came out to be statistically insignificant in a larger part of the estimation window, which can be explained by the lack of observed changes in the policy interest rates in the country.

In the case of the Indian economy, the *t*-test of the abnormal changes was found to be statistically insignificant which presented that the abnormal changes in the policy interest rates in the economy were not impacted by the changes in the real GDP as triggered by the outbreak of the Covid-19 global pandemic.

In the specific case for the Brazilian economy, it is evident from the student's *T*-test results that the changes in the policy interest rates were unaffected by the changes in the real GDP or by the announcement of the coronavirus outbreak by the World Health Organization.

The Chinese economy, as can be seen from the results of the *t*-test of abnormal changes, reflected statistically significant results. This allows us to decipher that a lack of variation in policy interest rates, as ought to be theoretically triggered by the changes in the real GDP, caused an abnormal pattern to arise in the policy interest rates in the current scenario. This abnormality can, however, be explained by the controlled economic structure in China, a notion which is evident in the constant policy rates exercised by the monetary authorities in the economy.

The results of the event study analysis can be simplified by describing a basic macroeconomic scenario which involves the announcement of a global pandemic which is likely to affect the existing labor pool and the productive capacity of the economy in the short-run. The impact of the outbreak being such that the production in the country slows down considerably, and the demand side of the economy is impacted by the limitations put in place by the policymakers. This scenario would lead to a deterioration of the real GDP of an economy in the months just after the announcement is made and the economy prepares to shut down. Subsequently, economies revoked the economic shutdowns, and the economy opens up. However, the government lowers the interest rates to propel the productive capacity and bring back the economy from its economic slump. This scenario describes the current market scenario rather closely, and the lowering of the policy interest rates by the major world economies presents itself as a piece of evidence for the monetary expansion sought by the governments worldwide. In summary, this study compares with the significance of the policy interest rate affected by real GDP among above five countries, we find that there is a significant difference between the change of policy interest rate and real GDP in the United Kingdom and China, however, the effect of real GDP of Japan, India, and Brazil on the change of policy interest rate is not significant. As the representatives of emerging market countries, such as China, Brazil, and India, the regional identification of policy interest rate is weaker than that of developed economies. The reason is that the policy interest rate system of emerging countries is immature, the degree of marketization is low, and the policy interest rate has not experienced a free and complete evolution process. In particular, since the outbreak of the Covid-19 epidemic, the economic situation of all countries in the world has been affected by the impact of the Covid-19 epidemic. Therefore, governments around the world have adopted more loose monetary policies to release monetary liquidity in order to strengthen the role of economic recovery. This abnormality can, however, be explained by the controlled economic structure in China, a notion which is evident in the constant policy rates exercised by the monetary authorities in the economy. Although the United Kingdom and Japan are both developed countries with a high degree of financial marketization, the main reasons why Japan's real GDP has no significant effect on policy interest rate are the high degree of aging, low labor productivity and the end of the credit boom. After the asset price bubble crisis, Japanese business departments and family departments were deeply in debt, leading to the rapid evolution of their optimization process from profit maximization to debt minimization, resulting in a shrinking demand for loans. Moreover, the life cycle characteristics of residents' asset liability structure tend to weaken the channels of monetary interest rate and credit, but strengthen the channels of wealth effect. Therefore, the effect of monetary policy in the aging economy ultimately depends on the relative importance of each transmission channel (26–29). Therefore, Japan's real GDP has no significant effect on the policy interest rate.

Considering that the theoretical change of a country's policy interest rate is always affected by some factors, such as fiscal policy, monetary policy, balance of payments, inflation, and so on. It is difficult to select one of these indicators to directly replace the theoretical change of policy interest rate. Therefore, this study follows the research method of Baker et al. (30) and uses the ”principal component analysis“ method to construct the policy interest rate index to reflect the theoretical change of policy interest rate as a whole. In this method, many indexes with certain correlation are recombined into a new group of comprehensive indexes by the way of principal component dimension reduction to replace the original indexes. As a result, this study selects industrial added value, net fiscal expenditure, balance of payments, foreign exchange reserves, M2 and unemployment rate as principal component analysis indicators, and constructs the theoretical change of policy interest rate index. Furthermore, this study selects the policy interest rate index instead of the initial theoretical change as the dependent variable to recalculate the above linear regression model. The results are as follows. The robustness test in Table A1 of Appendix results are basically consistent with the above conclusions, and it is proved that the estimation results are robust.

Overall, governments aim to get risk of adverse effects of Coronavirus outbreak on the economy by lowering their policy interest rates. In this period, lowering of the policy interest rates by the major economies is for stimulating the economic development and increasing the price level. This study suggests the changes in the monetary policy, as instigated by the unprecedented natural or even man-made events, are rather sporadic and cannot be expected to perform against the theoretical models constructed based on the historical observations of the macroeconomic variables like inflation and output.

## Conclusion

The monetary policy allows the policymakers to control the supply of money and credit in the economy. Taking two opposite directions, we have the two forms of monetary policies, expansionary and contractionary. Out of these two, the expansionary monetary policy is sought as a way out for the policymakers to pull the economy from a period of the economic slump through injecting credit into the economy and promoting economic growth within the economy. As the economies run along the course of business cycles, depression, and recovery is a part of their cyclical economic fluctuations, reducing and increasing policy interest rate relative to the changes in the real GDP and inflation is rather common. However, the cyclical fluctuations in the business cycles can be led astray by the occurrence of unprecedented events and/or natural disasters which affect the productive capacity of the economy in short-run, along with having long-run repercussions. One such event which surfaced on the world economic landscape was the outbreak of the coronavirus, in January 2020, as declared by the World Health Organization. The virus outbreak soon gained momentum and evolved into a global pandemic, which sent the policymakers into a flurry of safety procedures and several stringent measures were imposed including national lockdowns as well as travel restrictions and public curfews. The series of events that followed had a detrimental impact on the production and consumption levels in the economies worldwide. This event can be seen as a trigger for changes being made in the monetary policies worldwide to ensure that the productive capacity of the economies is not majorly affected and the economy does not fall to the abyss of economic recession.

This article examined five major economies of the world, United Kingdom, Japan, Brazil, Chin and lastly, India, for the changes in the monetary policy decisions that have been implemented following the Covid-19 outbreak. The assessment was undertaken in the form of an event study analysis, further substantiated with a regression analysis conducted for testing the significance of CPI and real GDP in predicting the policy interest rates in the economy. The results of the regression analysis produced significant results in each of the five economies, which then paved the way for the event study method to be implemented. The WHO issued a series of instructions to the world leaders against a possible virus outbreak originating from China on 10 January 2020, which served as the core event being examined and thereby allowing us to draw up an event window of 9 months ranging from 19 September till 20 May. The estimation window was framed over 20 years, spanning from January 2000 till May 2020. The impact was sought in the form of changes in the policy interest rates, relative to the changes observed in the real GDP as triggered by the announcement of the event. The results of the event study analysis presented that the abnormal changes in the interest rates were statistically significant in the case of the United Kingdom, Brazil, and China, while the abnormal changes were found to be statistically insignificant in the case of India and Japan. This leads to infer the abnormalities in the patterns of changes observed in the policy interest rates in these economies was not substantiated by the changes in the real GDP. In certain cases, the changes made in the interest rates were negligible, as was the case of China and Japan and in other cases the changes made to counteract the economic impact of the Covid-19 outbreak were sporadic. This presents us with the conclusion that the changes in the monetary policy, as instigated by the unprecedented natural or even manmade events, are rather sporadic and cannot be expected to perform against the theoretical models constructed based on the historical observations of the macroeconomic variables like inflation and output.

Further examining the long-standing economic impact of the Covid-19 crisis on the world economies cannot be yet fully examined as the event is still afresh and the event window is rather small and the post-event window being absent. It would be interesting to examine the state of the monetary policy changes, as triggered by the changes in the rates of inflation and real GDP, as triggered by the global pandemic by the end of 2020.

## Data Availability Statement

The original contributions presented in the study are included in the article/supplementary material, further inquiries can be directed to the corresponding author.

## Author Contributions

YL: writing, discussion, and analysis. YS: data analysis, writing, and supervision. MC: writing and data collection. All authors: contributed to the article and approved the submitted version.

## Conflict of Interest

The authors declare that the research was conducted in the absence of any commercial or financial relationships that could be construed as a potential conflict of interest.

## Appendix

**Table A1:** Robustness check.

	**UK**	**Japan**	**India**	**China**	**Brazil**
Intercept	6.3656 [3.7630]	−6.8371 [−6.7638]	15.5741 [4.8731]	−3.7798 [−1.5783]	110.7238 [6.8934]
CPI	−0.1576 [−32.8739]	0.0058^***^ [2.3870]	0.0193^**^ [2.9834]	−0.0369^**^ [−1.0039]	−0.1877 [−9.8933]
Real GDP	0.0583^**^ [5.8955]	0.0762^**^ [6.0324]	−0.2831 [−1.9032]	0.0379^**^ [2.4461]	−1.2291 [−7.9062]
R square	0.8723	0.3117	0.0589	0.0436	0.7099

*** *Implies that the probability value of the t-statistic of the regression coefficient is less than 0.01, making the coefficient statistically significant at 99% level of significance*.

** *Implies that the probability value of the t-statistic of the regression coefficient is less than 0.05, making the coefficient statistically significant at 95% level of significance*.

* *Implies that the probability value of the t-statistic of the regression coefficient is less than 0.10, making the coefficient statistically significant at 90% level of significance*.
